# Association between Diabetes Mellitus and Tuberculosis in United States-Born and Foreign-Born Populations in San Francisco

**DOI:** 10.1371/journal.pone.0114442

**Published:** 2014-12-05

**Authors:** Gompol Suwanpimolkul, Jennifer A. Grinsdale, Leah G. Jarlsberg, Julie Higashi, Dennis H. Osmond, Philip C. Hopewell, Midori Kato-Maeda

**Affiliations:** 1 Curry International Tuberculosis Center, University of California San Francisco, San Francisco, California, United States of America; 2 Faculty of Medicine, Chulalongkorn University and King Chulalongkorn Memorial Hospital, Thai Red Cross Society, Bangkok, Thailand; 3 Tuberculosis Control, San Francisco Department of Public Health, San Francisco, California, United States of America; 4 Department of Epidemiology and Biostatistics, University of California San Francisco, San Francisco, California, United States of America; Public Health Agency of Barcelona, Spain

## Abstract

**Setting:**

The impact of diabetes on tuberculosis in United States and foreign-born populations in San Francisco has not been studied.

**Objective:**

To determine the characteristics, prevalence and temporal trends of diabetes in US and foreign-born persons attending the San Francisco Tuberculosis Clinic.

**Design:**

We analyzed data from individuals seeking medical attention at the San Francisco Tuberculosis Clinic. We included patients with diagnosis of tuberculosis, latent infection, or not infected with *Mycobacterium tuberculosis*. We assessed the temporal trend and the characteristics of individuals with and without diabetes.

**Result:**

Between 2005 and 2012, there were 4371 (19.0%) individuals without evidence of tuberculosis infection, 17,856 (77.6%) with latent tuberculosis, and 791 (3.4%) with tuberculosis. 66% were born in the United States, China, Mexico, and the Philippines. The prevalence of diabetes was the highest among individuals with tuberculosis and increased during the study period. Patients with tuberculosis and diabetes were more likely to be male, older than 45 years and born in the Philippines. There was a disproportionate association of TB and DM relative to LTBI and DM among Filipinos in individuals older than 45 years old.

**Conclusions:**

Our data suggest that Filipinos older than 45 years old are more likely to have tuberculosis probably due to a higher prevalence of diabetes. In San Francisco, tuberculosis-screening programs in individuals with diabetes and latent tuberculosis may be beneficial in patients older than 45 years old especially from the Philippines.

## Introduction

The prevalence of diabetes mellitus (DM) among patients with tuberculosis (TB) increased in United States from 8% in 2010 [1] to 14% in 2012 [2]. Globally, it is estimated that 10% of TB cases are associated with DM [3]. A meta-analysis of cohort studies demonstrated that DM was associated with TB with a relative risk (RR) of 3.1 (95% confidence interval (CI) 2.3 to 4.3) compared with non-diabetic individuals, although in observational studies the odds ratios were highly variable [4]. Patients with DM and TB were more likely to have an unfavorable treatment outcome (treatment failure or death) (RR 1.7, 95%CI 1.4 to 2.1) and more likely to relapse (RR 3.9, 95%CI 2.4 to 6.2) than patients without DM [5]. Although there is no evidence that DM increases susceptibility to infection by *Mycobacterium tuberculosis* [6], DM is associated with alterations in inflammatory responses such as activation of the inflammation cascade that appear to induce and maintain the subacute inflammatory state associated with obesity [7].

The estimated prevalence of DM in persons 20 to 79 years old increased globally from 5.1% in 2003 to 8.3% in 2012 [8], [9]. It is predicted that the world prevalence of DM will increase from 285 million in 2010 to 439 million by 2030, with 36 million diabetics in the United States (US) alone [10]. In 2012, the estimated prevalence of DM in US in persons born in Mexico, the Philippines, China, and US was 15.6%, 9.6%, 8.8%, and 9.3%, respectively [9].

In 2010, 85% of the TB cases in San Francisco occurred in foreign-born persons, mainly from the Philippines, China and Mexico with an estimated incidence of 90, 32 and 23 cases per 100,000 persons, respectively, compared with an incidence of 2.8 per 100,000 persons among the US-born population [11].

We undertook this analysis to determine the prevalence and temporal trends of DM in three main groups attending the San Francisco Tuberculosis Clinic (SFTB clinic): persons with latent tuberculosis infection (LTBI), persons with TB and persons with no evidence of LTBI or TB. We also examined the clinical, epidemiological and bacterial characteristics of patients with TB with and without DM in the different US- and foreign-born populations. Among the bacterial characteristics, we also included the analysis of the lineage of *M. tuberculosis* [12], as there is evidence of variable inflammatory response by different lineages [13]. Some of the results of this study have been previously reported in abstract form at the American Thoracic Society 2012 International Conference [14].

## Materials and Methods

We performed a retrospective study of the data collected by the San Francisco Tuberculosis Control Section. We included information from all individuals seeking medical attention who had a final diagnosis of LTBI, TB or no evidence of LTBI or TB. We analyzed demographic and clinical information from all individuals as well as microbiological characteristics in patients with TB.

### Ethics statement

The study was approved by the University of California, San Francisco, Human Research Protection Program. Consent form was not obtained as the study was based on de-identified information collected as part of standard of care.

### Lineages of M.tb

[12] There are 7 lineages based on genomic regions of difference and single nucleotide polymorphisms [12], [15]. In this study, we used methods to determine the six lineages prevalent in San Francisco (the seventh lineage was described in Ethiopia and has not been detected in SF (data not shown)) [12], [15] The six lineages are: *M. tuberculosis* Indo-Oceanic lineage (or lineage 1), East Asian (or lineage 2), East African Indian (or lineage 3), Euro-American lineage (lineage 4), West African 1 (or lineage 5), and West African 2 (or lineage 6).

### TB categories

All individuals seeking medical attention in the San Francisco TB control section were classified in one of the following TB categories [16]:

- no evidence of *M. tuberculosis* infection (TB categories 0-no exposure to TB or 1-not infected based on the ATS classification [16]): no evidence of TB and negative tuberculin skin test or an interferon gamma release assay.
- LTBI (category 2 on the ATS classification [16]): positive tuberculin skin test or interferon gamma release assay and no evidence of TB.
- TB (category 3 on the ATS classification [16]) reported as a case of TB.

### Treatment outcome of TB

We included: completed treatment, treatment failure [17], default, transferred and death [17], [18].

### Diagnosis of DM

This was based on the information reported by the patient and medical records. During the study period, there was no change in screening policies for DM among individuals seeking care at the San Francisco TB control section.

### Statistical Analysis

The temporal trend of DM prevalence in the 3 TB categories was assessed using the Cochran-Armitage test. We compared the prevalence of DM within the three TB categories among different populations stratified by age by comparing odds ratios using Z-score. Clinical, epidemiological and bacterial characteristics were compared between TB patients with and without DM among the different US- and foreign-born populations using Chi-squared test and logistic regression. We included interaction terms to the regression model to determine interactions between diabetes and place of birth on TB versus LTBI. We used Wilcoxon rank-sum to test for differences between medians of treatment length and time to conversion of the sputum. Statistical analyses were performed with SAS version 9.2 (SAS Institute Inc., Cary, NC, USA).

## Results

### Study population

Between April 2005 and March 2012, 25,293 individuals seeking medical attention were evaluated for TB and LTBI. If treated for either TB or LTBI, management was usually conducted at the SFTB clinic. Of the total evaluated, 23,018 had a category determined: 4371 (19.0%) did not have evidence of *M. tuberculosis* infection, 17,856 (77.6%) had LTBI; and 791 (3.4%) had TB. Of those with a TB category determined, 6732 (29.2%) were born in the US, 3582 (15.6%) in China, 2530 (11.0%) in Mexico, 2544 (11.1%) in the Philippines, and 7630 (33.1%) in other countries (Table 1).

**Table 1:** Prevalence of DM in the different TB categories and in the different FB and US-born population, April 2005-March 2012.

Populations	DM in individuals with TB (%) [95%CI]	DM in individuals with LTBI (%) [95%CI]	DM in individuals without evidence of MTB infection (%) [95%CI]	OR (95%CI), p-value*	Z-score, p-value
All (n = 23,018)	126/791 (15.9%) [13.4–18.5%]	1158/17,856 (6.5%) [6.1–6.9%]	206/4371 (4.7%) [4.1–5.3%]		
Philippines (n = 2544)	36/113 (31.9%) [23.3–40.5%]	140/1815 (7.7%) [6.5–8.9%]	41/616 (6.7%) [4.7–8.6%]	5.59 (3.63–8.61), p<0.001	Reference
China (n = 3582)	40/233 (17.2%) [12.3–22.0%]	159/2569 (6.2%) [5.3–7.1%]	31/780 (4.0%) [2.6–5.4%]	3.14 (2.16–4.58), p<0.001	−1.974, p = 0.048
Mexico (n = 2530)	4/48 (8.3%) [0.5–16.2%]	144/2109 (6.8%) [5.8–7.9%]	11/373 (2.9%) [1.2–4.7%]	1.24 (0.44–3.5), p = 0.68	−2.627, p = 0.01
US (n = 6732)	16/173 (9.2%) [4.9–13.6%]	350/5391 (6.5%) [5.8–7.2%]	51/1168 (4.4%) [3.2–5.5%]	1.47 (0.87–2.48), p = 0.15	−3.855, p<0.001

*Comparison between DM in individuals with LTBI and DM in individuals with TB.

Z-score: compares odds ratio between different populations by using Philippines as a reference.

### Prevalence of DM

**Figure 1:**
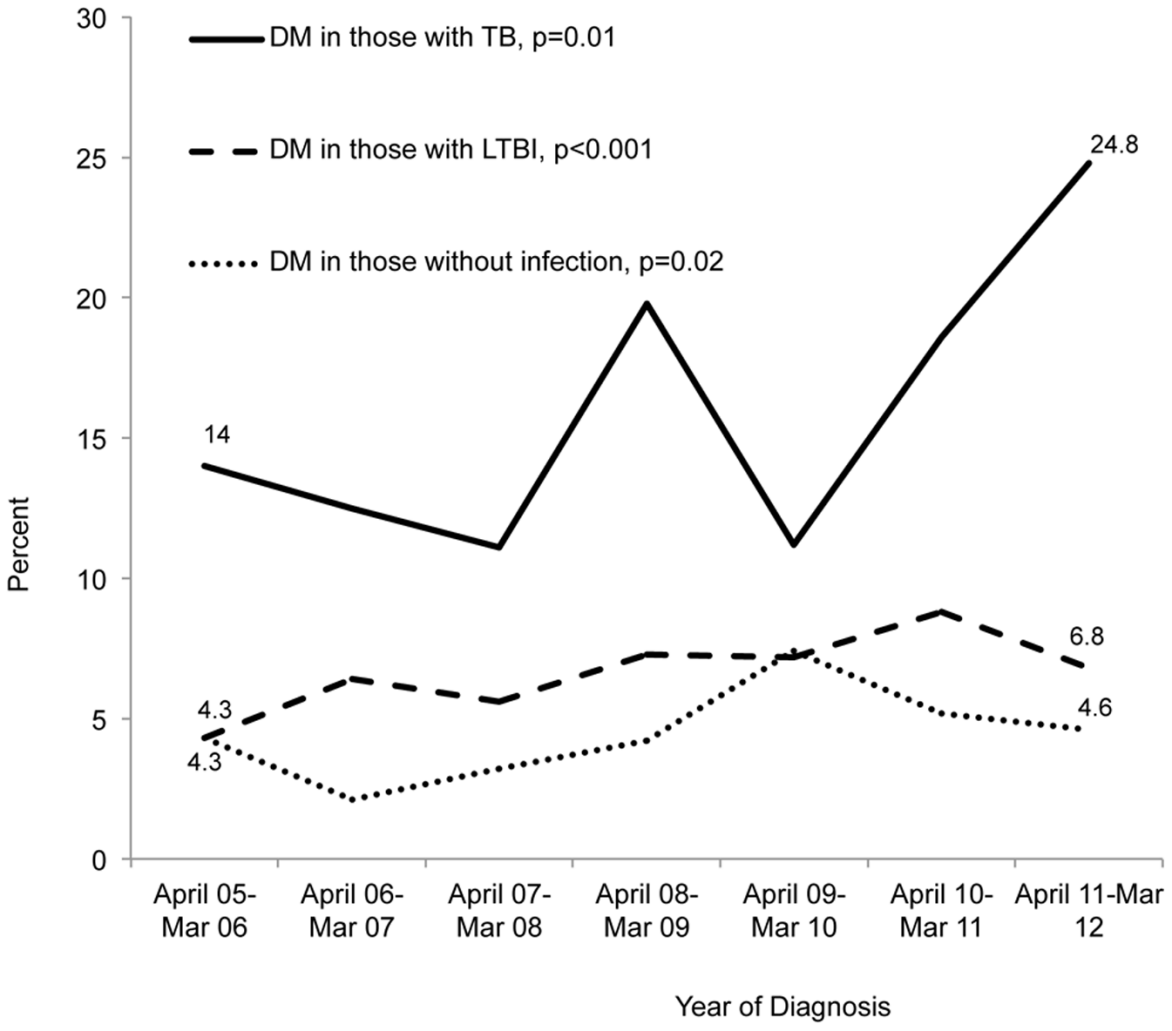
Prevalence of DM in the different TB categories in all study subjects: April 2005- March 2012. The temporal trend of DM was assessed using Cochran-Armitage test for trends.

**Figure 2:**
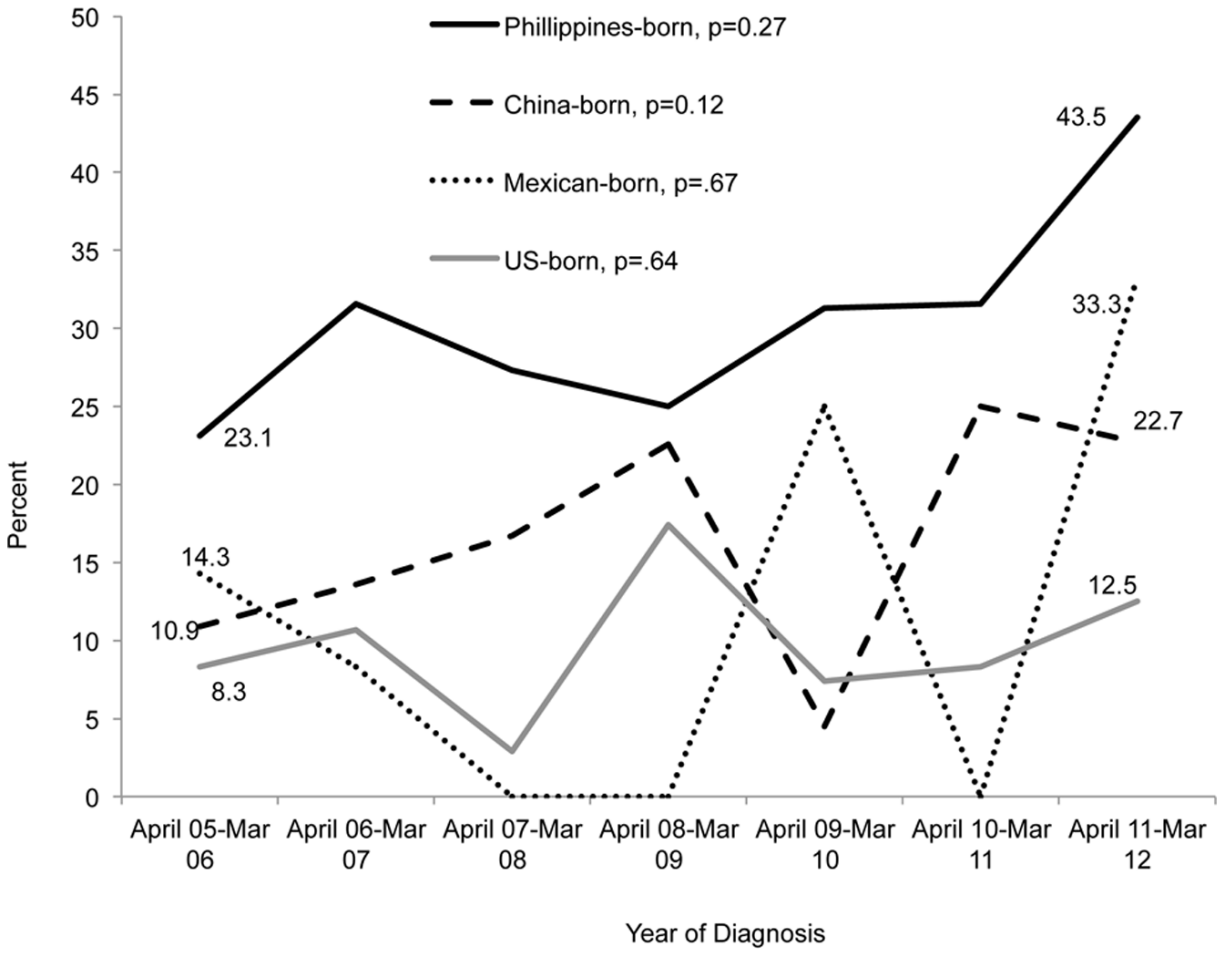
Prevalence of DM among TB patients in the US and foreign-born populations: April 2005- March 2012. The temporal trend of DM prevalence was assessed using Cochran-Armitage test for trends.

The overall prevalence of DM was highest among individuals with TB (126/791; 15.9%), followed by those with LTBI (1158/17,856; 6.5%) and by individuals without LTBI or TB (206/4371; 4.7%) (Table 1). The prevalence of DM in the different TB categories increased from April 2006 to March 2012, among patients with TB and known DM status, (p* = *0.01) specifically from 16/114 (14.0%) in the period from April 2005 to March 2006 to 30/121 (24.8%) in the period of April 2011 to March 2012 (Figure 1). During the same time period, the prevalence of HIV among TB patients decreased from 12/114 (10.5%) in 2005-06 to 5/121 (4.1%) in 2011-12 (p = 0.03). The prevalence of DM in patients with TB from the Philippines increased from 23.1% to 43.5% (p = 0.27). The prevalence for the other foreign and US-born groups was more variable (Figure 2).

The prevalence of DM in patients with TB varied from 31.9% (36/113) in individuals born in the Philippines to 8.3% (4/48) in those born in Mexico (Table 1). The prevalence of DM in individuals with LTBI varied from 7.7% (140/1,815) for those born in the Philippines to 6.2% (159/2,569) for those born in China; and the prevalence of DM in individuals without LTBI or TB varied from 6.7% (41/616) for those born in the Philippines to 2.9% (11/373) for those born in Mexico (Table 1). Among the US-born population with TB, the prevalence of DM varied depending on race/ethnicity: 60% (3/5) for American Indian, 33.3% (1/3) for Pacific Islanders, 13.6% (9/66) for African Americans, 4.5% (1/22) for Hispanics, 4.1% (1/25) for Asians, and 1.9% (1/52) for Whites.

Because the prevalence of DM increased with age we performed a stratified analysis (Table 2). In all TB categories, Philippines-born patients older than 45 years old had the highest prevalence of DM, especially among individuals with TB. The proportion of TB with DM relative to the proportion of LTBI with DM was significantly higher among the individuals from the Philippines when compared with the other populations (vs. US-born, Z-score =  −2.782, p = 0.002; vs. Mexican-born, Z-score =  −1.115, p = 0.13; vs. China-born, Z-score =  −2.333, p = 0.009) (Table 2).

**Table 2:** Prevalence of DM in the different TB categories and in the different FB and US-born populations, April 2005-March 2012 stratified by age.

AGE ≤ 45					
Populations	DM in individuals with TB (%) [95%CI]	DM in individuals with LTBI (%) [95%CI]	DM in individuals without evidence of MTB infection (%) [95%CI]	OR (95%CI), p-value*	Z-score, p-value
All	13/302 (4.3%) [2.0–6.6%]	301/10,867 (2.8%) [2.5–3.1%]	59/3097 (1.9%) [1.4–2.4%]	1.58 (0.90–2.78), p = 0.11	
Philippines	1/26 (3.9%) [0.0–11.2%]	31/1081 (2.9%) [1.9–3.9%]	6/387 (1.6%) [0.6–2.8%]	1.35 (0.18–10.3), p = 0.77	Reference
China	2/46 (4.4%) [0.0–10.2%]	19/1415 (1.3%) [0.7–1.9%]	9/496 (1.8%) [0.6–3.0%]	3.34 (0.75–14.8), p = 0.11	0.702, p = 0.24
Mexico	2/39 (5.1%) [0.0–12.1%]	78/1697 (4.6%) [3.6–5.6%]	9/340 (2.7%) [0.9–4.4%]	1.12 (0.27–4.74, p = 0.88	−0.148, p = 0.44
US	4/77 (5.2%) [0.2–10.2%]	74/2647 (2.8%) [2.2–3.4%]	10/754 (1.3%) [0.5–2.1%]	1.91 (0.68–5.35), p = 0.22	0.293, p = 0.38
AGE> 45					
All	113/489 (23.1%) [19.4–26.8%]	857/6989 (12.3%) [11.5–13.0%]	147/1274 (11.5%) [9.8–13.3%]	2.15 (1.72–2.69), p<0.001	
Philippines	35/87 (40.2%) [29.9–50.5%]	109/734 (14.9%) [12.3–17.4%]	35/229 (15.3%) [10.6–19.9%]	3.86 (2.40–6.20), p<0.001	Reference
China	38/187 (20.3%) [14.6–26.1%]	140/1154 (12.1%) [10.3–14.0%]	22/284 (7.8%) [4.6–10.9%]	1.85 (1.24–2.75), p = 0.002	−2.333, p = 0.009
Mexico	2/9 (22.2%) [0.0–49.4%]	66/412 (16.0%) [12.5–19.6%]	2/33 (6.1%) [0.0–14.2%]	1.50 (0.30–7.4), p = 0.62	−1.115, p = 0.13
US	12/96 (12.5%) [5.9–19.1%]	276/2744 (10.1%) [8.9–11.2%]	41/414 (9.9%) [7.0–12.8%]	1.28 (0.69–2.37), p = 0.44	−2.782, p = 0.002

*Comparison between DM in individuals with LTBI and DM in individuals with TB.

Z-score: compares odds ratio between different populations by using Philippines as a reference.

### Impact of place of birth on patients with TB and DM (Table 3)

During the study period, 791 patients were diagnosed with TB. One hundred twenty-six patients reported DM (15.9%). In order to determine if the place of birth was an independent factor associated with patients with TB and DM, we performed an adjusted analysis of the characteristics of patients with TB and DM and without DM (Table 3). We found that patients with TB and DM were more likely to be born in the Philippines (OR 2.29, 95%CI 1.41–3.72, p<0.001), to be male (OR 1.66, 95%CI 1.02–2.68, p = 0.04), and older than 45 years (OR 5.78, 95%CI 3.13–10.7, p<0.001). To determine if the effect of DM on TB and LTBI was similar in all the populations we tested for interactions. We found significant interaction (p<0.001) between Philippines birth and DM such that Filipinos with DM are much more likely to have active TB versus LTBI than other-born with DM (Table 4). There was no association between DM and isoniazid resistant or multi-drug resistant TB (Table 3). Lineage 1 strains were associated with DM. However, because patients from the Philippines are more likely to have TB due to lineage 1 strains [12], we performed a stratified analysis to determine if DM was associated with lineage 1 in patients born outside the Philippines. We did not find a significant association, although this was possibly due to a small sample size.

**Table 3:** Characteristics of tuberculosis patients based on their diabetes status.

Characteristic	Diabetes n (%)	No Diabetes n (%)	Unadjusted odds ratio (95%CI), p-value	Adjusted* odds ratio (95%CI), p-value
N	126 (15.9)	665 (84.1)		
Place of birth				
Philippines	36 (31.9)	77 (68.1)	3.05 (1.94–4.81), <.001	2.29 (1.41–3.72), <.001
Other	90 (13.3)	588 (86.7)		
Sex				
Male	96 (18.9)	413 (81.1)	1.95 (1.26–3.03), 0.002	1.66 (1.02–2.68), 0.04
Female	30 (10.6)	252 (89.4)		
Age				
> 45	113 (23.2)	375 (76.8)	6.72 (3.71–12.2), <.001	5.78 (3.13–10.7), <.001
≤ 45	13 (4.3)	290 (95.7)		
HIV† status				
Positive	3 (4.9)	58 (95.1)	0.25 (0.08–0.83), 0.02	0.36 (0.11–1.21), 0.10
Negative	87 (17)	426 (83)	Referent	Referent
Unknown	36 (16.6)	181 (83.4)	0.97 (0.64–1.49), 0.90	0.98 (0.61–1.56), 0.92
Sputum smear status				
Sputum smear positive	51 (21.0)	192 (79.0)	1.51 (1.01–2.27), 0.04	1.26 (0.79–2.02), 0.33
Sputum smear negative	67 (14.9)	382 (85.1)	Referent	Referent
No sputum or solely extrapulmonary	8 (8.1)	91 (91.9)	0.50 (0.23–1.08), 0.08	0.48 (0.20–1.11), 0.09
Cavities				
Pulmonary cavitary TB	23 (21.5)	84 (78.5)	1.50 (0.89–2.52), 0.13	1.53 (0.84–2.77), 0.16
Non cavitary	78 (15.5)	426 (84.5)	Referent	Referent
Solely extrapulmonary TB	25 (13.9)	155 (86.1)	0.88 (0.54–1.43), 0.61	1.36 (0.75–2.45), 0.31
Lineage‡				
Indo Oceanic	33 (26.6)	91 (73.4)	Referent	
East Asian	28 (16.8)	139 (83.2)	0.56 (0.31–0.98), 0.04	
Euro American	31 (13.6)	197 (86.4)	0.43 (0.25–0.75), 0.002	
Lineage specific‡				
Indo Oceanic lineage	33 (26.6)	91 (73.4)	2.07 (1.27–3.35), 0.003	
Non-Indo Oceanic lineage	59 (14.9)	336 (85.1)		
Philippine-born				
Indo Oceanic	22 (31.4)	48 (68.6)	Incalculable	
East Asian	0 (0)	2 (100)		
Euro American	0 (0)	3 (100)		
Non-Philippine-born‡				
Indo Oceanic	11 (20.4)	43 (79.6)	Referent	
East Asian	28 (17)	137 (83)	0.80 (0.37–1.74), 0.57	
Euro American	31 (13.8)	194 (86.2)	0.62 (0.29–1.34), 0.23	
Any isoniazid resistance				
Isoniazid resistant	12 (18.2)	54 (81.8)	1.07 (0.55–2.07), 0.85	
Isoniazid susceptible	93 (17.3)	446 (82.8)		
Multi-drug resistance				
Multi-drug resistant	0 (0)	8 (100)	Incalculable	
Non-multi drug resistant	105 (17.6)	492 (82.4)		

*Adjusted for: sex, age>45 years, HIV status, birth in the Philippines, AFB smear status, and cavitary/pulmonary TB.

† HIV = Human immunodeficiency virus.

‡ Eight patients had East African Indian lineage, and 1 had West African-1; none of these had diabetes.

**Table 4:** Interaction terms for DM and place of birth as predictors of TB and LTBI, adjusted for age.

Characteristic	TB n (%)	LTBI n (%)	Unadjusted odds ratio (95%CI), p-value	Adjusted odds ratio (95%CI), p-value
N	791	17,856		
Age				
> 45	489 (6.5)	6989 (93.5)	2.52 (2.17–2.92), <.001	2.18 (1.86–2.54), <.001
≤ 45	302 (2.7)	10,867 (97.3)		
Diabetes				
Present	126 (9.8)	1158 (90.2)	2.73 (2.24–3.34), <.001	1.81 (1.37–2.39), <.001
Absent	665 (3.8)	16,698 (96.2)		
Place of birth				
Philippines	113 (5.9)	1815 (94.1)	Referent	Referent
China	233 (8.3)	2569 (91.7)	1.46 (1.15–1.84), 0.001	1.67 (1.27–2.19), <.001
Mexico	48 (2.2)	2109 (97.8)	0.37 (0.26–0.52), <.001	0.58 (0.40–0.85), 0.005
Other foreign	224 (3.6)	5972 (96.4)	0.60 (0.48–0.76), <.001	0.81 (0.61–1.06), 0.12
US	173 (3.1)	5391 (96.9)	0.52 (0.40–0.66), <.001	0.62 (0.47–0.82), <.001
Interaction terms				
Philippines x diabetes				Referent
DM	36 (20.5)	140 (79.6)	5.59 (3.63–8.61), <.001	
No DM	77 (4.4)	1675 (95.6)		
China x diabetes				0.56 (0.31–0.99), 0.05
DM	40 (20.1)	159 (79.9)	3.14 (2.16–4.58), <.001	
No DM	193 (7.4)	2410 (92.6)		
Mexico x diabetes				0.23 (0.08–0.72), 0.01
DM	4 (2.7)	144 (97.3)	1.24 (0.44–3.50), 0.68	
No DM	44 (2.2)	1965 (97.8)		
Other x diabetes				0.41 (0.23–0.75), 0.003
DM	30 (7.6)	365 (92.4)	2.38 (1.59–3.54), <.001	
No DM	194 (3.3)	5607 (96.7)		
US x diabetes				0.29 (0.15–0.57), <.001
DM	16 (4.4)	350 (95.6)	1.47 (0.87–2.48), 0.15	
No DM	157 (3.0)	5041 (96.9)		

TB treatment outcome was reported in 735 of 791 cases (92.9%) and were similar between the 116 patients with DM and 619 without DM: 97 (83.6%) and 538 (86.9%) respectively completed treatment, 4 (3.4%) and 26 (4.2%) transferred out and 1 (0.9%) and 7 (1.1%) defaulted. The frequency of death was higher among patients with DM (14 [12.1%]) than in patients without DM (48 [7.6%]) but it was not statistically significant (OR 1.63, 95%CI: 0.87–3.07, p = 0.13). There were no treatment failures. The treatment outcome was also similar between the US and different foreign-born populations (data not shown). Patients with TB and DM received longer treatment with a median of 9.0 months (interquartile range (IQR) 6.5–10.3) compared with 6.7 months (IQR 6.2–9.5) in patients without DM, (p = 0.001). Data of the time of the conversion of sputum was available only in 66 of 126 (52.4%) patients with TB and DM and in 283 of 665 (42.6%) patients with TB and without DM. The median weeks to convert to negative sputum was 6.1 weeks (IQR 4.6–9.0) for those with DM and 5 weeks (IQR 3.7–8.4) for those without DM (p = 0.04).

## Discussion

Due to the parallel epidemics of TB and DM, in 2011 the World Health Organization (WHO) made the recommendation that TB surveillance should be performed among patients with DM in settings where the TB incidence was more than 100 cases per 100,000 inhabitants [6]. They recommended testing for DM among TB patients in all countries. Due to the limited resources available to most TB control programs and the increasing number of patients with DM, it is very likely that these recommendations have not been followed in most countries despite the convergence of the two epidemics.

There has not been any systematic screening strategy during the study period that could have resulted in a relatively higher proportion of patients from the Philippines in San Francisco with DM. The increase of DM in the different TB categories during the study period can be explained by the increase of DM in the overall population. In San Francisco, the overall prevalence of DM increased from 3.8% in 2001 [19] to 6.2% in 2009 [20]. An increase in the overall prevalence of DM has also being reported in Mexico from 7.4% in 2003 to 15.9% in 2012, in China from 2.7% in 2003 to 8.82% in 2012 and in the Philippines from 3% in 2003 to 9.65% in 2012 [8], [9]. The overall incidence of TB in San Francisco among the Philippine-born individuals is also higher (90 per 100,000) than among individuals born in China-, Mexico- and US (32, 23 and 2.8 per 100,000 population, respectively) [11].

The clinical and epidemiological characteristics of the patients with TB and DM varied among the different populations. Patients with TB and DM born in the Philippines and China were older than 45 years old when compared to patients with TB without DM. A similar trend was observed in patients from the US and Mexico but it was not statistically significant. The association of TB and DM was statistically significant in patients from the Philippines. In addition, we observed an interaction between DM and being born in the Philippines comparing TB to LTBI patients that was significantly greater than the interaction with any other country of birth. Although these are cross-sectional data and we therefore cannot attribute causality, the data suggest that persons with DM born in the Philippines may progress from LTBI to TB at a higher rate than persons born elsewhere. A higher progression rate could be due to biological factors, such as the lineage of TB found in patients from the Philippines, or it could be due to behavioral and social factors, such as poorer control of DM, or some combination of factors. As described before, more than 80% of the TB in patients from the Philippines was due to *M. tuberculosis* strains from lineage 1, one of the ancient lineages, which have a distinctive inflammatory response [13] when compared with the other lineages frequently observed in San Francisco. We are unable to test the hypothesis that *M. tuberculosis* lineage may be a factor associated with TB and DM because lineage is so highly correlated with place of birth in our patients that we have no power to examine it. How the bacterial factors associate with the immunopathogenesis of TB and DM will be explored in further studies. A recent publication that included insured patients with diabetes type 2 from the San Francisco Bay Area showed that patients from the Philippines (and India) had significantly worse control measured by HbA1c value in spite of access to healthcare [21].

In contrast with several reports [5], [22], [23], [24], we did not find statistically significant differences in the outcome of TB treatment in patients with and without DM, probably due to a small sample size to detect differences. However we did see a trend of higher mortality, longer time to sputum conversion and longer treatment in patients with TB and DM compared with patients with TB and without DM. The level of glycemic control was not available to determine if this was associated with the outcome of TB treatment as reported in other studies [24].

Our study has the following limitations. First, our database did not include variables associated with the prevalence of DM such as body mass index and socioeconomic status. However, based on a recent study in San Francisco, the frequency of obesity is lower among the Asian population (7.1%) when compared with the other ethnic groups: 13.3% in Whites, 33.4% in African American and 56.9% in Latino [25]. Lower socioeconomic status has been also associated with DM [26], [27], [28] and TB [29], [30]. However, based on the San Francisco Department of Public Health data individuals born in the Philippines were less likely to belong to the lower socioeconomic status (defined by frequency of unemployment, poverty status, uninsured rates and public high school graduation) than Latino or Chinese populations [25]. Second, the definition of diabetes was based on self-report and the medical record, which may underestimate the actual prevalence of DM. However, a community-based study in the US demonstrated that the prevalence of self-reported diagnosis of DM was similar to self-reported DM combined with clinical and laboratory evaluations [31], and has shown to have reasonable validity for public health surveillance [32], [33]. Third, we did not have information about the degree to which patients with DM were controlling their DM.

## Conclusions

The prevalence of DM in patients with TB has increased significantly in the past 7 years in San Francisco. Data suggest that among the different populations, Filipinos older than 45 years old were more likely to have TB probably due both to a higher prevalence of DM and to a higher probability of progressing from LTBI to TB. Therefore, in San Francisco, in spite of a relatively low incidence of TB, TB screening programs and preventive therapy should be considered for individuals with DM, specifically in patients more than 45 years old and especially in older patients from the Philippines. Although we did not find a negative outcome in patients with DM and TB, DM screening programs in patients with TB will be important. Prospective studies of DM among TB patients are needed to confirm this observation.
